# Does Case-Finding for Admission to Aged Care Rapid Investigation and Assessment Unit for Older Patients Improve Hospital Length of Stay? *Evaluation of ARIA Unit*

**DOI:** 10.5334/ijic.7038

**Published:** 2023-10-16

**Authors:** Sundhar R. Balu, Angela Khoo, Carol Lu Hunter, Danielle Ní Chróinín

**Affiliations:** 1 Department of Geriatric Medicine, Liverpool Hospital, Liverpool, AU; 2 Department of Geriatric Medicine, Shoalhaven District Memorial Hospital, Nowra, AU; 3 South Western Sydney Clinical School, UNSW Sydney, Liverpool, AU

**Keywords:** admission length, emergency geriatrics, representation

## Abstract

**Introduction:**

Many older people present to emergency departments annually, often with complex geriatric syndromes, yet current acute care models and traditional admissions process may under-serve their needs. The multidisciplinary Aged Care Rapid Investigation and Assessment (ARIA) Unit seeks to bridge this gap, by actively identifying and assessing patients.

**Methods:**

A prospective case-control study was undertaken at a single-centre tertiary referral institution. Patients were eligible for inclusion in ARIA group if admitted to ARIA via case-finding by the geriatrician or Aged Care Services Emergency Team, whilst standard geriatric admissions formed the control group. This study evaluates whether ARIA reduced hospital length-of-stay (LOS) and representation rates.

**Results:**

370 patients were included (185 each arm) with similar baseline demographics, frailty scores, and Charlson Comorbidity Indices. Patients admitted to ARIA had significantly shorter hospital LOS than those via standard pathway (3.3 days [IQR2.2–5.8] vs 7.5 days [IQR4.2–13.7], p < 0.00001). There were no significant differences in 90-day representation rates (n = 66 [35.7%] vs n = 64 [34.6%], p = 0.82).

**Discussion/Conclusion:**

Introduction of an ARIA unit with a targeted approach to frontline geriatric services and case-finding is associated with improved LOS of older acute hospital patients. An economical cost analysis of this study would be beneficial in exploring potential financial savings.

## Introduction

Australians are living longer and healthier lives, with individuals aged ≥65 years contributing 15% of Australia’s population in 2017, with anticipated increase to 22% by 2057 [1]. Across 2016–2017, there were 7.8 million presentations to emergency departments (ED), averaging 21,000 daily. Patients aged ≥65 years accounted for 21% of these and are thereby over-represented compared to their proportion of the general population [2]. This is compounded by the fact that older patients often require longer ED stays due to complex medical dispositions requiring extensive investigations [3]. Older patients presenting with geriatric syndromes such as delirium, dementia, falls, polypharmacy, and frailty are further predisposed to higher rates of hospital representation [4]. The current care model in many EDs is not tailored to cater for specific care needs of older patients, necessitating development of strategies to aid clinical decision-making and improve outcomes [5,6].

Nolte and Pitchforth [7] postulated that integrated care could be attained by “overcoming issues of fragmentation through linkage or coordination of services of different providers along the continuum of care”. As such, the gaps seen in timely delivery of acute geriatric care might be bridged by joint ventures between ED and aged care services which enable access to comprehensive geriatric assessment of frail older people to swiftly identify complex geriatric issues and devise integrated management plans [8]. One such strategy is a geriatrician-led case-finding model-of-care, whereby rather than waiting for review by an ED clinician and subsequently geriatrician referral, older patients in ED are identified from patient dashboards and seen first by a geriatrician. Evidence suggests that such a model may reduce hospital admission rates and improve longer-term management [9,10]. Beyond ED, in the inpatient setting, multidimensional and interdisciplinary assessments of frail older patients’ biopsychosocial care needs, combined with integrated treatment plans, reduce mortality and increase community dwelling status at 12 months post-admission [11]. However, models are rarely generalisable to all scenarios, and local factors like socioeconomic status, health literacy, and availability of community support services may influence the required structure [9,10].

In this context, we developed the Aged Care Rapid Investigation and Assessment (ARIA) unit in February 2018 in a tertiary hospital in Sydney, Australia (described below). This unit consisted of a dedicated team involved in active case-finding from ED presentations, as well as a ward space for those requiring brief admissions that provided accelerated access to essential multidisciplinary care. The objectives of this study were to determine whether, compared to previous standard geriatric admissions processes, patients admitted to ARIA unit via an active case-finding system had: (1) reduced hospital length of stay (LOS), (2) reduced ED LOS, and (3) avoided increases in hospital representations within 90 days of index discharge. We hypothesised that ARIA implementation would improve LOS without increased 90-day representation.

### Problem Statement

Despite the high numbers of older people presenting to EDs, and complex nature of presentations in this group current models-of-care do not provide optimal care within this acute setting. The introduction of an Aged Care Rapid Investigation and Assessment team (ARIA), with a targeted approach to front-line geriatric services and active case-finding direct from ED presentations, may improve hospital length-of-stay for older acute hospital patients without increasing representation rates. Key elements of this model-of-care can then be translated into wider clinical setting sand improve care for a broad cohort of patients.

## Research Methods

### Study Design

We conducted a prospective case-control study at a tertiary hospital in Sydney, Australia, to determine outcomes from implementation of a case-finding system and admissions of older persons to a geriatric multidisciplinary short-stay unit, namely ARIA. Data collection occurred in the setting of routine review by geriatricians to inform service planning, research, and quality improvement, and not solely for this project.

The ARIA model-of-care consists of both a physical ward space and a dedicated team comprising a geriatrician, advanced and basic physician trainee, junior medical officer, and full allied health service (physiotherapy, occupational therapy, social work, speech pathology, dietitian). The ARIA team actively identifies and assesses patients prior to ED referral via twice-daily meetings and patient dashboard review together with the Aged Care Services Emergency Team (ASET). Screening for potentially suitable patients includes reviewing triage notes and identifying keywords such as “falls” or “delirium”. This allows identification of ED patients who would benefit from rapid upfront multidisciplinary geriatric assessment, diagnostics, and management, and bypasses the need for ED clinician review prior to geriatrics review. The ARIA team can thereby either discharge older patients home directly from ED with appropriate follow-up in place, or admit those that require inpatient management either to the dedicated short-stay ARIA unit, or, if more appropriate, to geriatric home wards. For the purposes of defining the ARIA intervention in this study, only patients who are reviewed by the ARIA team in ED and subsequently admitted to the physical ARIA short-stay unit are included. On-site cover is provided during weekday business hours and half-day on weekends.

Once admitted into the dedicated ARIA ward, the bulk of the intervention consists of dedicated, intentional, and daily multidisciplinary input (in contrast to general ward settings where multidisciplinary input maybe sporadic and less coordinated). The patient journey includes daily consultant-led ward rounds (in contrast to general ward settings where consultants round 2-3 times per week). This is supplemented by daily allied health reviews with blanket referrals being made to physiotherapy, occupational therapy, and social work services. Complementing these are twice-daily journey board meetings to ensure patients receive accelerated access to necessary investigations and development of integrated care plans. The hospital’s patient flow coordinator assists with expediting investigations for patients within the ARIA unit. The structure of the ARIA ward round and follow-up includes daily family updates, and liaison with general practitioners upon discharge from hospital to ensure safe transition back to the community.

Patients suitable for ARIA include older patients with atypical, complex multisystem presentations and/or ‘traditional’ geriatric syndromes [4]. The team then identifies the patient’s medical, psychosocial, and functional limitations to develop a coordinated plan, maximising the patient’s recovery by providing early mobilisation, rehabilitation, and comprehensive discharge planning. Notably, patients with behavioural disturbances, including extreme aggression or exit-seeking behaviour, are excluded from ARIA admission, as the unit is not a secured facility.

### Ethics Approval

Approval as a low to negligible risk (LNR) study was obtained from the South Western Sydney Local Health District human research ethics committee. Application was made for waiver of consent on the basis that this study carried low risk with likely patient benefit to be gained, using routinely collected aged care data, and the consent process would have delayed the patient flow process between emergency department and wards, thereby potentially negatively impacting upon patient care. The waiver of consent thereby permitted use of deidentified patient data. This approval was obtained on 20^th^ January 2004; no reference number was provided at this time.

### Eligibility Criteria

Inclusion criteria for ARIA group were patients aged ≥65 years, who were actively case-found by a geriatrician or ASET team, with index admissions between February 2018 and March 2019. Patients initially admitted to ARIA and subsequently transferred to a geriatrics ward were included. Patients discharged directly from ED were excluded.

Inclusion criteria for control group were patients aged ≥65 years, who underwent geriatrics admissions via standard pathway prior to existence of ARIA, with index admissions between December 2016 and January 2018. Given the greater number of such presentations, random selection of an equivalent number of patients to the ARIA interventional group was generated using computer-based randomisation.

Residential status at time of admission (community-based vs residential aged care facilities [RACF]) did not affect eligibility for either arm.

### Definitions

Triage category was that assigned by ED at arrival [12], with a high triage category defined as 1 or 2. Baseline frailty scores were assigned per the Canadian Study of Health and Ageing (CSHA) [13] and morbidity burden per the Charlson Comorbidity Index (CCI) [14]. Culturally and linguistically diverse (CALD) population was defined as people originating from countries where English was not the primary language [15], and non-English speaking (NES) status applied to those unable to communicate their medical history in English.

### Outcomes

Primary outcome was to assess whether implementation of a case-finding model-of-care for admission of older persons to ARIA unit influenced likelihood of long LOS (i.e. LOS within the highest quartile) compared to standard geriatric admission processes.

Secondary outcomes included assessing whether this model-of-care influenced ED LOS and representation rates within 90 days (of index discharge).

### Statistical Analysis

Statistical analyses were performed using Stata V13.0 (StataCorp, Texas, USA). We performed descriptive analyses of patient characteristics and outcomes before and after ARIA implementation. Categorical variables were compared using Chi-square or Fisher’s exact test, as appropriate, and continuous data using T-test or Mann Whitney U test. Univariate and multivariate regression analyses evaluated variables potentially associated with outcomes of interest.

Variables were included in multivariable model if an association was observed on univariate analysis (p < 0.05), as per a priori strategy. Model-building was performed using forward stepwise logistic regression. If there was statistical evidence of collinearity between candidate predictor variables, these would not be included in the final model if inclusion did not change the model fit (as assessed by pseudo *R*^2^) or findings.

LOS was measured both as a binary outcome (prolonged vs not), and a continuous variable in sensitivity analysis.

## Results

### Patient Characteristics

A total of 185 patients were case-found for ARIA admission (interventional group). For the non-ARIA group (control group), there were 1106 admissions under geriatric medicine in the study period. Of these, we randomly selected an equal number of patients (n = 185) as control arm. A total of 370 patients were thus included. No data were missing, and no patients lost to follow-up.

There was no difference in baseline characteristics between ARIA and non-ARIA groups (Table 1), including age, sex, CALD/NES status, CSHA frailty status, and residential status (all p ≥ 0.1).

**Table 1:** Demographic and clinical characteristics (n unless otherwise specified).


CHARACTERISTICS	ARIA	NON-ARIA (CONTROL)	P-VALUE

Age (median)	85.6 years	84.5 years	0.1

Female sex	91:94	77:108	0.1

CALD	107	120	0.2

Non-English-Speaking	70	74	0.7

Residence (Community:RACF)	158:27	146:39	0.1

CSHA Frailty scale			0.2

*Category 1*	0	1	

*Category 2*	0	1	

*Category 3*	0	3	

*Category 4*	9	14	

*Category 5*	60	68	

*Category 6*	84	65	

*Category 7*	31	33	

CCI (median [IQR])	6 (5–7)	6 (5-8)	0.2

Dementia	60	63	0.8

Delirium	50	73	0.008

Active BPSD	6	13	0.1

Triage Category			0.003

*Category 1*	1 (0.5%)	1 (0.5%)	

*Category 2*	28 (15.2%)	46 (24.9%)	

*Category 3*	151 (82.0 %)	123 (66.5 %)	

*Category 4*	3(1.6%)	15 (8.1%)	

*Category 5*	1 (0.5%)	0 (0.0%)	

High Triage Category (1+2)	29 (15.7%)	47 (25.4%)	0.02


There were significantly more patients with delirium in control group compared to ARIA group (39.5% vs 27.0%, p < 0.01), but similar proportions of patients with dementia (p = 0.8) and behavioural/psychological symptoms of dementia (BPSD) (p = 0.1). Degree of comorbidities was similar between groups (non-ARIA CCI 6 [IQR5-7] vs ARIA CCI 6 [IQR5-8], p = 0.2). Most patients were triage category 3 (n = 274/370, 74.1%), but there were more high triage category patients (category 1/2) in the control group (p = 0.02).

### Primary Outcomes

#### Total Hospital LOS

Patients in ARIA group had significantly shorter hospital LOS of 3.3 days (IQR2.2–5.8) compared to those admitted via standard admissions pathway (LOS 7.5 days, IQR4.2–13.7, p < 0.001).

**Figure 1:**
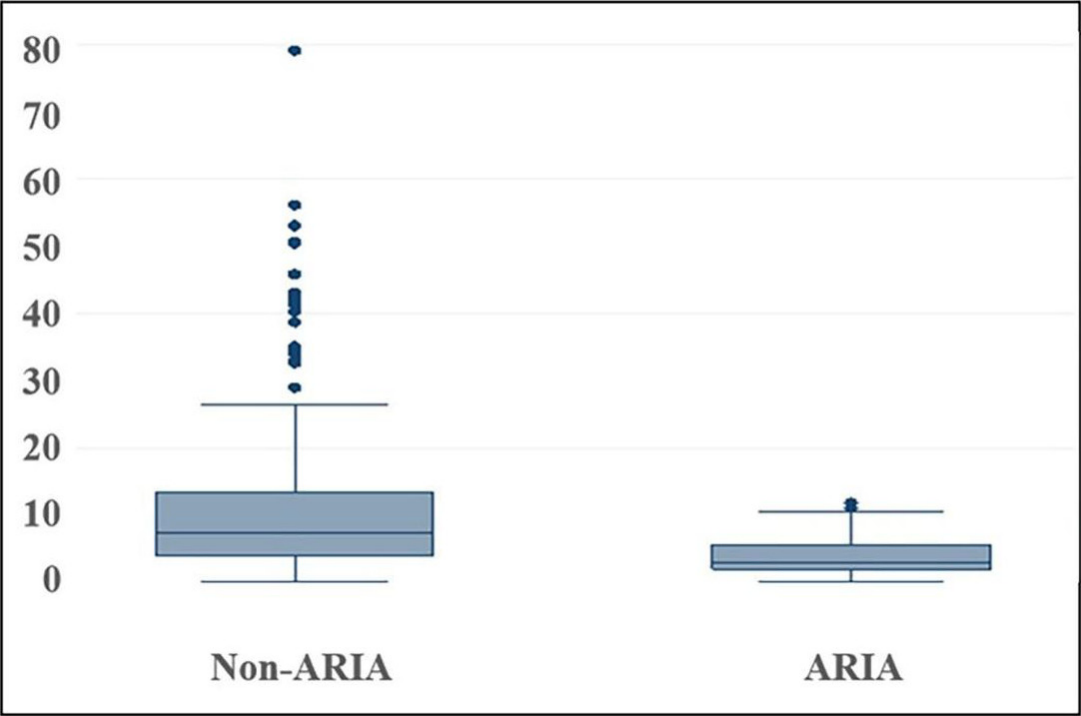
Boxplot of LOS according to ARIA versus non-ARIA (unadjusted).

Long LOS was defined as LOS in the top quartile (>9.2 days) (Figure 1). ARIA admission was associated with lower odds of long LOS (OR0.1, 95%CI 0.1–0.3, p < 0.001). Factors associated with long LOS on univariate analysis included community domicile (OR4.7, 95%CI 1.6–13.4, p < 0.004), whilst an inverse association was noted with increasing age (OR0.9 per unit increase, 95%CI 0.9–1.0, p < 0.001) and CCI (OR0.8, 95%CI 0.7–0.9, p < 0.003). On multivariate logistic regression analysis, adjusting for age, CCI, and community domicile, ARIA status was independently associated with lower odds of long LOS (OR0.1, 95%CI 0.04–0.2, p < 0.001) (Table 2).

**Table 2:** Analysis of factors potentially associated with hospital length of stay. CSHA = Canadian Study of Health & Aging. CCI = Charlson Comorbidity Index.


UNIVARIATE ANALYSIS			

VARIABLE	ODDS RATIO	95% CI	P-VALUE

ARIA	0.1	0.1–0.3	<0.0001

Age	0.9	0.9–1.0*	<0.001

Female Sex	1.0	0.6–1.6	0.9

Dementia	1.2	0.7–2.1	0.5

Delirium	1.0	0.6–1.7	1.0

CSHA Frailty	0.9	0.7–1.2	0.6

CCI score	0.8	0.7–0.	<0.003

Community Domicile	4.7	1.6–13.4	<0.004

High Triage Category	0.7	0.4–1.4	0.34

**MULTIVARIATE ANALYSIS**			

***VARIABLE***	***ODDS RATIO***	***95% CI***	***P-VALUE***

ARIA	0.1	0.04–0.2	<0.0001

Age	1.0	0.9–1.0*	<0.02

CCI score	0.9	0.8–1.0	0.1

Community Domicile	6.1	2.1–18.2	<0.001


* Discrepancy between confidence interval and p-value occurs due to rounding up to one decimal place.

Findings were similar when LOS was examined as a continuous variable. On multivariate linear regression analysis, ARIA maintained a weak association with hospital LOS (coefficient –7.1, 95%CI –14.5–0.3, p = 0.1).

In a sensitivity analysis, to determine if findings were driven by the lower proportion of patients with delirium or higher triage category at arrival (Table 1), we excluded (1) those with delirium and then (separately) (2) those with high ED triage category. Findings were unchanged when only including those without delirium (3.4 vs 7.4 days, p < 0.001), or only those with lower triage category (3.4 vs 7.7 days, p < 0.001).

### Secondary Outcomes

#### ED LOS

We did not observe any difference in ED LOS (time from triage to ward admission) between ARIA and control groups (7.6 hours [IQR4.9–12.9] vs 8.0 hours [IQR5.4–11.9], p = 0.6).

Long ED LOS was defined as ED LOS in the top quartile (>11.9 hours). On univariate analysis, ARIA status was not associated with lower odds of long ED LOS (OR1.1, 95%CI 0.7–1.8, p = 0.7), with similar multivariate analysis results (Table 3).

**Table 3:** Multivariate analysis of factors potentially associated with emergency department length of stay.


VARIABLE	ODDS RATIO	95% CI	P-VALUE

ARIA	1.1	0.6–1.7	0.9

Age	1.0	0.9–1.0*	<0.02

Dementia	0.7	0.4–1.2	0.2

Delirium	0.5	0.3–0.9	<0.02

Community Domicile	1.9	0.9–4.2	0.1


* Discrepancy between confidence interval and p-value occurs due to rounding up to one decimal place.

#### 90-Day Representation Rates

We did not observe any difference in 90-day hospital representation between ARIA and control groups (n = 64/185 [34.6%] vs n = 66/185 [35.7%] respectively, p = 0.8). ARIA status was not associated with increased odds of 90-day representation on both univariate (OR1.1, 95%CI 0.7–1.7, p = 0.7) and multivariate analysis (OR0.9, 95%CI 0.6–1.4, p = 0.6).

## Discussion

In this study exploring impact of a specialist geriatrician-led case-finding model-of-care, patients admitted to ARIA experienced reduced hospital LOS, without increased 90-day representation.

The average LOS for case-found patients (3.3 days) was half that of the control group (7.5 days). We noted a significant, independent association between ARIA status and reduced long LOS, even after adjusting for age, comorbidities, and residence. Encouragingly, these were achieved without increased representation rates by 90 days. Furthermore, for those who were readmitted, average time interval between index discharge and readmission was similar between groups.

We also hypothesised that ARIA status would be associated with lower likelihood of long ED LOS, however no significant difference was identified. We did note that delirium was independently, inversely associated with long ED LOS. Patients with delirium have specific care needs that are challenging to meet in a busy ED environment [4], and thus their transfer to a more suitable ward may be prioritised. Although we did not find association between ARIA admission and reduced ED LOS in this study, strategies previously suggested include regular and early case-finding by senior clinicians, or early treatment plan and discharge coordination by geriatric teams [9,16,17]. Additional extraneous contributing factors to ED LOS would also require evaluation, including institutional factors such as hospital bed capacity and types (e.g. isolation rooms), and staffing limitations.

Surprisingly, there was no difference in baseline presence of BPSD, dementia, and comorbidities. Whilst non-ARIA group exhibited slightly higher acuity in triage category, this was not associated with LOS. We noted independent relationship between community-dwelling status and likelihood of long LOS. We hypothesise this might be due to deficits in community-based supports (compared to RACFs), and/or the need to return to higher baseline functional status prior to discharge back to community settings.

We also noted inverse relationship between higher LOS and CCI, persisting even after adjusting for RACF residence. One proposition is that those with complicated morbidity opted for less aggressive goals of care and thus could be discharged more rapidly. However, we did not formally evaluate care limitations or functional status, and further exploration of these potential confounders would be beneficial.

Limited studies have focused on multidisciplinary assessments in ED and impacts on long LOS, with varied results. A UK study investigated a twelve-bed “comprehensive older person’s evaluation” zone within ED [18] and found this targeted approach to front-line geriatric services improved service delivery (at least up to one month follow-up) without impeding patient flow. Contrastingly, another similarly structured UK study [19] demonstrated an increase in long LOS, particularly amongst those aged >85 years. This was potentially because this model-of-care resulted in only the sickest patients being admitted [19]. Our study expanded upon this further by assessing longer-term outcome data and exploring major prognostic markers like comorbidities.

Nevertheless, our findings must be interpreted within light of limitations. This was a single-centre study, with moderate sample size. Safe patient discharge is dependent upon community resource availability, home situation, and presence of family/friend supports, which varies between settings. Therefore, our results cannot necessarily be extrapolated to other population groups or locations. While we endeavoured to adjust for pertinent variables, we may not have adjusted for all confounders, and we admit the possibility of patient selection ‘bias’ given our unit’s exclusion criteria, and some differences in patient characteristics. However, our findings were similar even after excluding delirious patients or the most acutely unwell.

Additionally, we utilised commonly reported healthcare outcomes [3,5] such as LOS and readmission rates, noting also that readmission may be a marker of poor quality care [20]. We acknowledge that these only address one aspect of a model’s success, and we did not formally gather data regarding patient and carer perspective and achievement (or otherwise) or patient-prioritised goals, although some provided feedback voluntarily. Platforms, such as the co-designed Health Outcomes and Patient Experience (HOPE), which is being implemented on a phase basis across New South Wales, Australia, will enhance our understanding of the lived experience for these patients [21].

In the absence of a ‘gold standard’ against which to benchmark our findings, we believe our data identifies a potentially beneficial new model-of-care, which may offset gaps in care of older patients provided by traditional models-of-care. We note that medical assessment units that provide early assessment and multidisciplinary care have associated reductions in length-of-stay amongst adult patients (up to 0.5-day reduction nationally [22] and 2.5-day internationally [23]). As we have built upon this model with the addition of case-finding and specialty-specific input, we believe that the observed benefits are likely real. We also note that even if an element of selection bias existed, included patients still appear to have benefited from reduced LOS compared to the cohort admitted prior to ARIA’s institution.

Study strengths include prospective data collection and capture of factors like CALD/NES status, frailty, comorbidity, and presence of geriatric syndromes. We performed consecutive patient sampling for ARIA group, which is considered the best amongst non-probability samples [24], whilst comparator group patients were randomly selected from within a consecutive sample. Additionally, analysing over one year’s worth of discharge data provides good representation of geriatric admissions across all seasons. We believe that the outcomes assessed are likely to be advantageous to clinicians, patients, and service planners alike.

Additionally, through restructuring, and without significant additional fiscal investment, the ARIA unit has been resourced with medical and allied health services. The associated reduced hospital LOS is not only important at a direct cost-savings level, but also been shown to minimise hospital-acquired complications and improves overall mortality and morbidity [25]. Whilst we did not perform in-depth economic analysis, we estimate that the observed difference in LOS even just for the 185 ARIA patients in this study equates to savings of approximately $1.2 million for our institution, based on costings for typical geriatric admissions (local data).

Enhancing community services and building on our model to facilitate transition even better across the hospital/community sector is likely to benefit patients further. Community services, however, are often area-dependent. Existing nation-wide services such as the Transitional Aged Care Program [26] promote post-hospitalisation rehabilitation, but access can be delayed pending approval process and/or availability of a position in the local program. Whilst some hospital-in-the-home models offer allied health input, our local version offers a more medicalised model, with nursing and physician input alone. Our wider aged care team recently piloted a multidisciplinary outreach program for older people presenting to the ED and/or ARIA, entitled “Care in the Community” to support safe return home and improve outcomes such as function and mobility. Early pilot data (not yet published in peer review journal) are encouraging [27], and we plan to build on this model, funding permitting.

## Conclusion

The introduction of ARIA unit with a targeted approach to front-line geriatric services and case-finding can be associated with reduced LOS for older acute hospital patients. Upscaling of the model-of-care could also improve care for wider cohorts of patients. ARIA model’s key elements of early identification, rapid access to investigations, streamlined specialist multidisciplinary assessment and management, and supported transition back to the community, should be translatable to other settings. Implementation will be best served if these key elements are adapted to local strengths and resources, and implementation embraced as a dynamic, evolving process.
